# Prevalence and molecular characterization of glucose-6-phosphate dehydrogenase deficiency in the Lue ethnic group of northern Thailand

**DOI:** 10.1038/s41598-021-82477-w

**Published:** 2021-02-03

**Authors:** Suwapat Sathupak, Kamonlak Leecharoenkiat, Jatupol Kampuansai

**Affiliations:** 1 Department of Biology, Faculty of Science, Chiang Mai University, Chiang Mai, Thailand; 2 Graduate School, Chiang Mai University, Chiang Mai, Thailand; 3 Oxidation in Red Cell Disorders Research Unit, Department of Clinical Microscopy, Faculty of Allied Health Sciences, Chulalongkorn University, Bangkok, Thailand; 4 Research Center in Bioresources for Agriculture, Industry and Medicine, Chiang Mai University, Chiang Mai, Thailand

**Keywords:** Genetic variation, Genetic variation, Haematological diseases

## Abstract

Glucose-6-phosphate dehydrogenase (G6PD) deficiency is one of the most common enzyme disorders. Prevalence and variant distribution of G6PD deficiency can vary in different regions and among differing ethnic groups. To reveal the G6PD frequency and molecular characterization among the Lue ethnic group of northern Thailand, blood samples of 296 unrelated individuals collecting from 6 Lue villages were analyzed. The observed G6PD enzyme activity ranged from 0.11 to 20.60 U/g Hb. Overall, 13.51% (40/296) of the individuals were identified as having G6PD deficiency status. The prevalence in males was 14.28% (20/140), while that of females was 12.82% (20/156). The most common G6PD variants in the Lue were the Kaiping 1388G > A (5.40%) and Canton 1376G > T (6.42%) types. Observed prevalence and variant types of the *G6PD* gene in the Lue population are similar to that of the Tai-Kadai speaking ethnic groups in southern China, which is consistent with their historically close line of ancestry. However, the founder effect that occurred during the Lue’s transboundary migration from China to Thailand showed its impact upon different patterns of G6PD distribution among each Lue village.

## Introduction

Glucose-6-phosphate dehydrogenase (G6PD) deficiency is an X-linked recessive hereditary red blood cell disorder. It is the most common genetic abnormality with an estimated 400 million people believed to be affected worldwide^1^. *G6PD* gene mutations can cause reductions in G6PD enzyme stability and activity. As a result, G6PD deficient red blood cells are at risk of oxidative stress destruction when triggered by infections, stressors, or the intake of some foods and drugs^2,3^. Most individuals with G6PD deficiency are clinically asymptomatic, but some *G6PD* mutations could lead to mild and severe manifested phenotypes such as neonatal jaundice, acute or chronic hemolytic anemia, neonatal hyperbilirubinemia, and/or favism^4,5^. There have been at least 140 point mutation in the *G6PD* gene that have been detected by molecular characterization globally^6^.

Distribution and predominant types of G6PD variants were found to be related to specific geographic locations and associated with different ethnic groups^7^. In Southeast Asia, a high prevalence of G6PD deficiency was particularly evidenced in the populations that reside along the Greater Mekong Subregion (GMS), which include China’s Yunnan Province, as well as areas in Cambodia, Vietnam, Laos, Myanmar, and Thailand. Malaria, which has historically been endemic in this region, is considered to be a major evolutionary force that is responsible for the high frequency of G6PD deficiency^8–10^. Different malaria parasite species and geographical separations have caused differences in the specificity of *G6PD* mutation types to certain ethnic groups^11^. For example, G6PD Mahidol (487G > A) is considered the predominant variant among Burmese^12^ and Kachin^13^ ethnic groups, while G6PD Viangchan (871G > A) is the most common in Thai^10^, Lao^9^ and Cambodian^14^ populations. Migration is another micro-evolutionary force that can drive the distribution of genetic traits and shape the similarity of the structure of populations by following the gene flow process^15^. Ethnic migrations have been recorded throughout the history of Southeast Asia ever since the prehistoric period, and these migrations have continued into the present days^16–18^. Although there have been a number of ethnic-based studies on the prevalence of G6PD deficiency in the GMS region, the influence of migration on the distribution of this red blood cell disorder has not yet been described. This study is the first investigation of the ethnic-based prevalence of G6PD deficiency among the Lue people who migrated across the upper GMS during the last few hundreds of years.

Lue is one of the Tai-Kadai speaking ethnic groups whose original homeland was in Xishuangbanna Dai autonomous prefecture, Southern China^19^. During the last millennium, the Lue confederated states regularly fell into military conflict among the Chinese, Burmese, Lanna and Siam (later Thailand) kingdoms. This resulted in the forcible movement of the Lue across the region as recruits, prisoners of war, tributary labourers, or slaves. The main stream of the Lue’s southward migration occurred from the late eighteenth century and lasted into the early nineteenth century by way of the Siamese (Thai) forced resettlement campaign^20^. In later generations, the Lue transboundary migration across the upper GMS has continued, driven by various political, cultural, and environmental factors. Owing to the movement that took place throughout their history, the Lue had eventually resettled in Myanmar, Laos, and Thailand. There are many Lue villages located in several provinces of Northern Thailand. Most of them still maintain their unique culture, tradition and dialect, while obvious differences emerge between ethnic groups^21^.

## Materials and methods

### Subjects

During the research, we protect the rights of participants and their identity and we confirmed that all experiments were performed in accordance with relevant guidelines and regulations based on the experimental protocol on human subjects which was approved by the Human Experimentation Committee of the Research Institute for Health Sciences, Chiang Mai University, Thailand. Written informed consents were obtained prior to the interview and blood sample collection.

**Figure 1:**
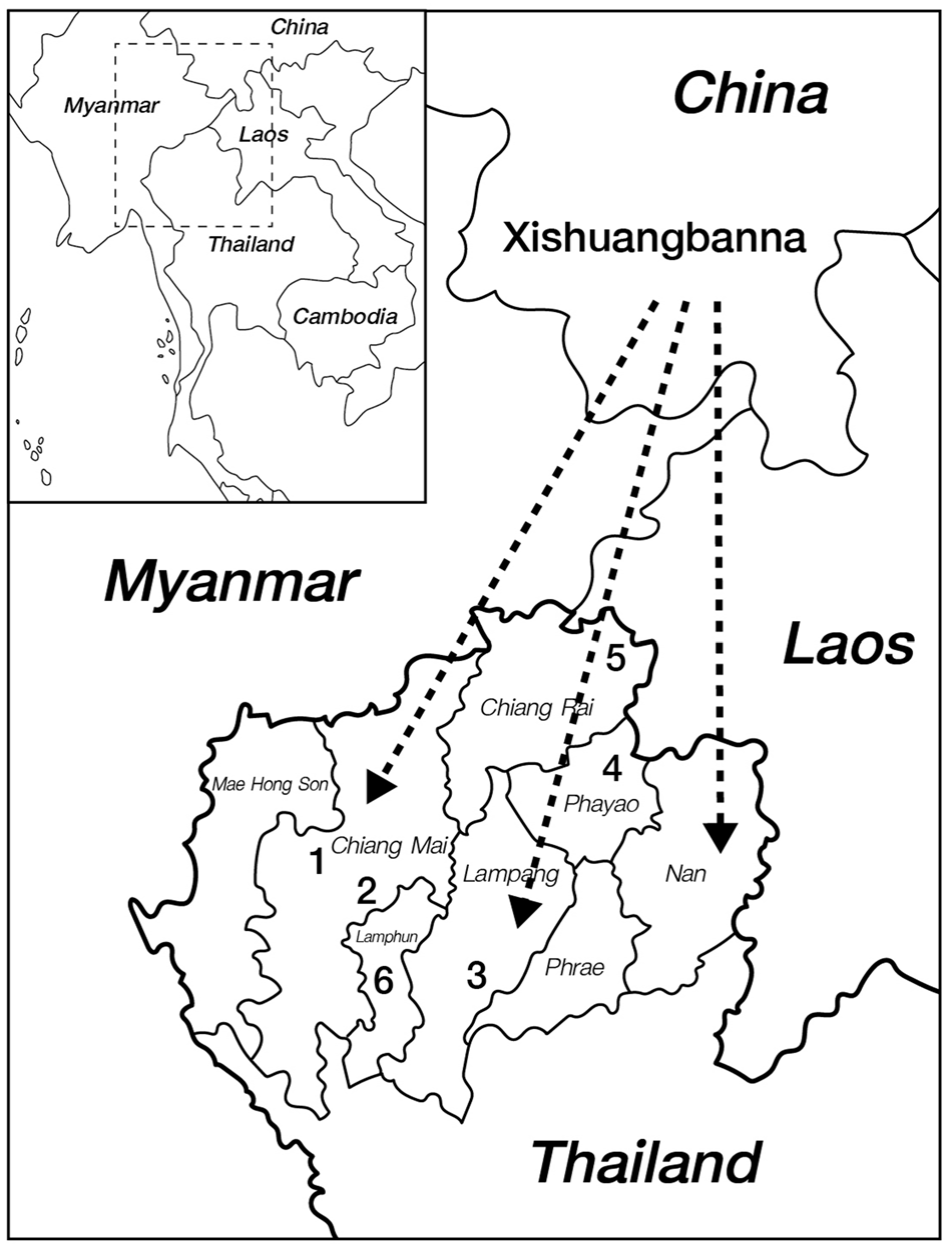
Schematic direction of the Lue’s migration from Southern China to Northern Thailand and the localities of studied villages. Drawing was adapted and used under license from Shutterstock.com. Original drawings can be found at https://www.shutterstock.com/image-vector/southeast-asia-map-565754383, https://www.shutterstock.com/image-vector/high-quality-map-northern-region-thailand-1664140510.

A total of 296 unrelated subjects (140 males and 156 females) residing in six Lue villages (Fig. 1 and Table 1) were enrolled. Volunteers were healthy subjects who were over 20 years old, of Lue ethnicity and who had no ancestors that were known to be from other recognized ethnic groups for at least three generations. Personal data were collected by research staff using form-based oral interviews of subjects for self-reported unrelated lineages, linguistics, migration histories and hematological diseases. Five millilitres of peripheral blood samples were collected from each individual using anticoagulant vacutainers. Blood samples were immediately stored at 4–8 °C until further analysis.

**Table 1:** General data, G6PD deficiency prevalence and typing of studied subjects.

Code	Lue1	Lue2	Lue3	Lue4	Lue5	Lue6	Total
Locality (district, province)	Samoeng, Chiang Mai	San Pa Tong, Chiang Mai	Mueang, Lampang	Chiang Kham, Phayao	Chiang Khong, Chiang Rai	Pa Sang, Lamphun	
Latitude (°N)/longitude (°E)	18°51′09"/98°41′54"	18°32′43"/98°51′4"	18°12′28"/99°29′26"	19°29′28"/100°16′13"	20°08′59"/100°24′43"	18°28′35"/98°56′03"	
No. of samples (M/F)	39 (16/23)	48 (23/25)	55 (28/27)	50 (26/24)	47 (24/23)	57 (23/34)	296 (140/156)
No. of deficiency cases (M/F)	7 (3/4)	4 (3/1)	8 (5/3)	8 (6/2)	3 (0/3)	10 (3/7)	40 (20/20)
No. of variant carriers (M/F)	8 (3/5)	7 (3/4)	14 (5/9)	11 (6/5)	5 (0/5)	13 (3/10)	58 (20/38)
Canton	1 (1/0)	3 (1/2)	7 (3/4)	5 (2/3)		3 (0/3)	19 (7/12)
Union	4 (0/4)		2 (1/1)			1 (1/0)	7 (2/5)
Viangchan	1 (0/1)					2 (1/1)	3 (1/2)
Mahidol		1 (1/0)					1 (1/0)
Kaiping		3 (1/2)	5 (1/4)	1 (1/0)	5 (0/5)	2 (0/2)	16 (3/13)
Coimbra						1 (0/1)	1 (0/1)
Valladolid/1311C > T	2 (2/0)						2 (2/0)
Aures				2 (1/1)			2 (1/1)
Gaohe				3 (2/1)			3 (2/1)
Unknown variant						4 (1/3)	4 (1/3)

### G6PD enzyme activity assay

Within 24 h after collecting, EDTA blood samples were measured for complete blood count by automatic hematology analyzer at PCT Laboratory Service, Bangkok, Thailand. G6PD enzyme activity was measured at the Faculty of Allied Health Sciences, Chulalongkorn University, Bangkok, Thailand, using Randox G-6-PDH reagent (Randox Laboratories Ltd, UK), following the manufacturer's instructions. In brief, 200 µL of packed red blood cells were isolated from 3 mL of EDTA whole blood by centrifugation (1500*g* 5 min) and washed three times in normal saline solution. The washed red blood cells were then lysed in the digitonin lysis buffer. The hemolysate was then incubated with the Glucose-6-phosphate substrate and cofactor NADP. Enzyme activity was determined by measurement of the rate of absorbance change at 340 nm at 37 °C using a RX Daytona analyser (Randox Laboratories Ltd, UK). G6PD enzyme activity values were ultimately reported in units per gram of Hb (U/g Hb) using the following formula:

$$\mathrm{G}6\mathrm{\text{PD activity U}}/\mathrm{\text{g Hb}}=\frac{\mathrm{\text{mU erythrocytes per ml}}\times 100}{\mathrm{\text{Hb }}(\mathrm{g}/\mathrm{dL})}\times {10}^{-3}$$

when 100 is the factor to convert from ml to dl, Hb (g/dl) is the hemoglobin concentration determined for each specimen, 10^−3^ is the factor to convert from mU to U.

During the course of measurement of enzyme activity, the Randox G-6-PDH normal and deficient controls were run in parallel with the tested samples according to the manufacturer's recommendations for quality control. Enzyme measurements were repeated twice for the G6PD deficient samples.

### G6PD mutation analyses

Genomic DNA was extracted using the inorganic salting out method^22^. We used a DiaPlexC™G6PD genotyping (Asian type) kit (SolGent, Daejeon, Korea) to screen all DNA samples for eight different *G6PD* variants; Vanua Lava (383 T > C), Mahidol (487G > A), Mediterranean (563C > T), Coimbra (592C > T), Viangchan (871G > A), Union (1360C > T), Canton (1376G > T), and Kaiping (1388G > A), according to the manufacturer’s protocol using the Eppendorf 6331 Nexus Gradient MasterCycler Thermal Cycler. Samples that had been found to display G6PD-deficient enzyme activity, but for which no variants could be identified using a genotyping kit, were then direct sequenced for their 12 *G6PD* exon sequences using primers modified from Nuchprayoon et al.^23^ (Supplementary Table 1). The PCR reaction of exon direct sequencing was carried out in 30 μl reaction containing 1X PCR buffer, 0.5 U of KOD-plus Neo *Taq* polymerase (Toyobo Co. Ltd., Japan), 5 ng of each primer, 1 mM MgSO_4_, 200 μM dNTPs, and 20–30 ng of DNA template. The optimized PCR conditions were employed at 94 °C for 2 min, following 40 cycles of 98 °C for 10 s, the annealing temperature for 30 s and an extension at 68 °C for 30 s. The PCR amplicons were direct sequenced at Macrogen Corp., South Korea. The exon direct sequencing technique was also performed on all G6PD variant female carrier to establish their homozygous/heterozygous condition.

### Statistical analysis

The Adjusted Male Median (AMM) G6PD activity (100% G6PD activity) was calculated to identify the cut-off values for G6PD deficiency by applying the calculation method described previously^24,25^. The AMM was defined as the median G6PD activity of all male participants after excluding samples with less than 10% of the overall median activity. Severe, moderate, and mild G6PD deficiency levels were defined as < 10%, < 30%, and < 60% G6PD activity of the AMM, respectively. The subjects who displayed G6PD activity at < 60% of the AMM were identified under the G6PD deficiency conditions, while individuals with ≥ 60% of the AMM were defined as G6PD normal.

Allelic frequency of each G6PD variant type was calculated using a simple counting scheme. Deviations from Hardy–Weinberg Equilibrium (HWE) among female genotypes were estimated by the Chi-square test using GraphPad Quickcalcs online tools (GraphPad Software, San Diego, USA).

Data on the G6PD deficiency prevalence of various ethnic groups that were enrolled in previous studies were retained for the purposes of comparison (Supplementary Table 2). Each group was classified by their spoken languages as Tai-Kadai, Sino-Tibetan, Austroasiatic, and Hmong-Mien linguistic families^26^. Prevalence of G6PD deficiency among different populations was compared using Fisher’s exact test, as implement in GraphPad Quickcalcs online tools (GraphPad Software, San Diego, USA).

## Results

### G6PD enzyme activity

G6PD enzyme activities of 296 Lue individuals ranged from 0.11 to 20.60 U/g Hb, with an average of 7.82 ± 3.73 U/g Hb. There were no significant differences in the average enzyme activity between genders with 7.47 ± 3.98 U/g Hb in males and 8.14 ± 3.47 U/g Hb in females, respectively (p > 0.05). The AMM enzyme activity was 7.80 U/g Hb, while 10%, 30%, and 60% of the AMM cut-off levels for severe, moderate, mild deficiency were 0.78, 2.34, 4.68 U/g Hb, respectively. Although, all participants in our survey were apparently healthy (with no self-reported hematological diseases and no sign of severe anemia on the day of blood collection), 40 subjects (13.51%) exhibited < 60% G6PD activity of the AMM cut-off level and were identified under the G6PD deficiency conditions (Table 1). Among these, 18 subjects (16 males and 2 females) who revealed enzyme activity lower than 0.78 U/g Hb were considered to have severe deficiency. There were 4 subjects (3 males and 1 female) who exhibited a moderate degree of deficiency, whereas 18 individuals (1 male and 17 females) exhibited a mild degree of deficiency. The average activity in the G6PD deficient males (0.68 ± 0.62 U/g Hb) was significantly lower than that of the G6PD deficient females (3.17 ± 1.24 U/g Hb).

### Identification of G6PD variations

Fifty-eight subjects (19.59%, 58/296) were determined to be carrying *G6PD* variants when investigated by DiaPlexC™G6PD Genotyping kit (Asian type) and direct sequencing methods (Table 2 and Supplementary Table 3). Forty samples (20 males and 20 females) exhibited enzymatic deficiency, whereas 18 heterozygous females showed normal activity (Table 2). There were 9 G6PD variation types, i.e. Canton (1376G > T), Union (1360C > T), Viangchan/Jammu (871G > A), Mahidol (487G > A), Kaiping (1388G > A), Coimbra (592C > T), Valladolid (406C > T), Aures (143 T > C), and Gaohe (95A > G), detected in the Lue population (Table 2). Of these, the Canton and Kaiping variants were the most predominant, occurring in 6.42% (19/296) and 5.40% (16/296) of the samples, respectively (Table 2). Mahidol and Coimbra types were relatively rare and were detected in only one subject each. The silent mutation 1311C > T in exon 11 co-occurred with Valladolid in 0.68% (2/296) of the tested individuals. However, we did not type for 1311C/T mutations in individuals who were variation-identified by the DiaPlexC™G6PD Genotyping kit. Thus, G6PD Viangchan (871G > A and 1311 T) and G6PD Jammu (871G > A, 1311C) could not be distinguished and the 871G > A mutation carriers were classified as G6PD Viangchan/Jammu as has been reported in this study. Genotype distributions among the female samples who carried each of the G6PD Kaiping, Union, Viangchan/Jammu, and Canton variants were in the HWE, while those of G6PD Gaohe and Aures deviated from the equilibrium (p < 0.0001) (Table 2). Note that HWE was not evaluated for G6PD Valladolid and Mahidol variants because they were only present in males. There were four subjects (1.35%) that exhibited a mild G6PD deficiency condition but did not harbor mutations at any of the genotyped sites.

**Table 2:** Prevalence of G6PD variants in Lue ethnic group.

G6PD variants	Normal G6PD activity			G6PD deficiency			Prevalence (%)	Allelic frequency	HWE p-value
	Male	Female		Male	Female				
	Hemizygotes	Homozygotes	Heterozygotes	Hemizygotes	Homozygotes	Heterozygotes			
**WHO class II**									
Kaiping			7	3		6	5.40	0.0270	0.2146
Union			2	2		3	2.36	0.0118	0.7621
Coimbra						1	0.34	0.0017	0.8106
Valladolid/1311 T				2			0.68	0.0034	
**WHO class III**									
Gaohe				2	1		1.01	0.0068	< 0.0001
Aures				1	1		0.68	0.0051	< 0.0001
Mahidol				1			0.34	0.0017	
Viangchan/Jammu						2	0.68	0.0034	0.6543
Canton			9	7		3	6.42	0.0321	0.6904
Unknown variant				1		3	1.35		
Total	0	0	18	20	2	18	19.59		

Among G6PD variants detected in this study, Kaiping, Union, Valladolid, and Coimbra were classified as Class II variants (causing severe deficiency) while Canton, Mahidol, Viangchan, Aures, and Gaohe were classified as Class III variants (causing moderate-to-mild deficiency) according to the World Health Organization’s classification on the level of enzyme activity^27^ (Table 2). Twenty-six Lue subjects who carried Class II variants displayed enzymatic activity ranging from 0.39 to 10.75 U/g Hb (Supplementary Table 3). Among these, six male subjects exhibited severe deficiency except for one case with the G6PD Kaiping variant who showed moderate deficiency (1.72 U/g Hb). Class II deficient females were comprised of the following breakdown; one severe deficiency, nine mild deficiency and nine normal activity cases. There were 28 participants who harbored Class III variants and displayed 0.11 to 14.35 U/g Hb enzymatic activity. Eleven Class III males displayed severe deficiency except for one who was a moderate deficient (1.51 U/g Hb) subject with G6PD Mahidol. Class III females consisted of five mild deficient cases and nine with normal activity. Interestingly, we observed two Class III homogygous females who exhibited different levels of enzyme activity. A G6PD Goahe homozygote female had severe deficiency (0.48 U/g Hb), while another G6PD Aures homozygote subject displayed a moderate degree of deficiency (1.34 U/g Hb).

### G6PD prevalence among Lue villages

Based on enzyme activity, the prevalence of G6PD deficiency was the highest in the Lue1 population (17.95%, 7/39), while the lowest was recorded in the Lue5 population (6.38%, 3/47). Furthermore, the highest observed number of G6PD variant carriers was found in the Lue3 population (Table 1). Additionally, the number of G6PD variation types was relatively highest (6 types) in the Lue6 group, while there were 1–4 types found in other villages. The G6PD Canton variant revealed the highest number of variation types in the Lue3 and Lue4 populations, while the Union variant was acknowledged as the highest type present in the Lue1 population. The G6PD Kaiping variant type was found within most of the Lue villages ranging from 2.00% (1/50) in Lue4 to 10.64% (5/47) in Lue5, but this type was missing in the Lue1 population. The Kaiping type was, moreover, the only G6PD variation found in the Lue5 population. G6PD Mahidol and Coimbra variants were found only in the Lue2 and Lue6 populations, respectively (Table 1).

**Figure 2:**
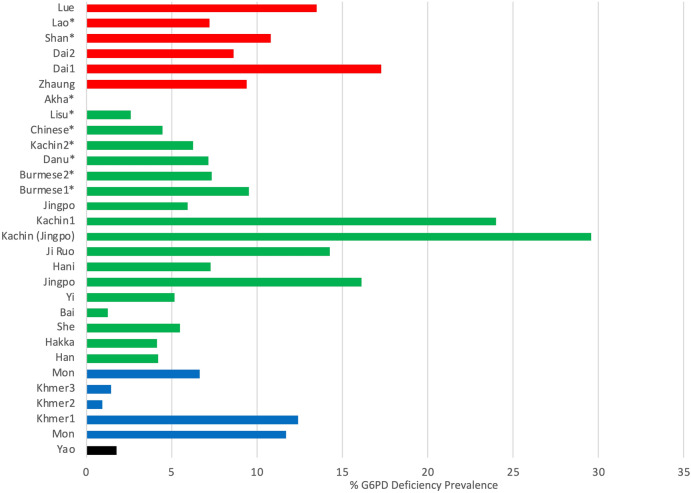
Prevalence of G6PD deficiency among ethnic groups residing in the upper Greater Mekong Subregion. Each ethnic group was classified by their spoken language as Tai-Kadai (red), Sino-Tibetan (green), Austroasiatic (blue), and Hmong-Mien (black) linguistic families. (*data obtained from males only).

Ethnic-based comparisons among the ethnic groups residing in the upper GMS revealed that the overall prevalence of G6PD deficiency in the Lue (13.51%) ethnic group was the same as the average level of the Tai-Kadai (9.69%) linguistic speakers, but was significantly higher than those of the Sino-Tibetan (4.51%), Austroasiatic (7.58%), and Hmong-Mien (1.77%) groups (p < 0.01). Note that the prevalence fluctuated among the ethnic groups who belonged to the Sino-Tibetan (1.25–29.55%) and Austroasiatic (0.97–12.42%) linguistic families. The prevalence among the Tai-Kadai speaking group was distributed in a relatively narrow range from 7.22% in the Lao people to 17.26% in the Dai ethnic group (Fig. 2).

## Discussion

Several studies have shown that the distribution of different G6PD deficiency prevalence levels and variants is related to geographical regions and ethnic groups^28^. Historically, the GMS has been an area that is vulnerable to malarial endemic outbreaks, and G6PD deficiency has long been recognized as a common red blood cell heredity disorder in this region^7,11^. Here, we evaluated the prevalence of G6PD deficiency among the Lue ethnic people who migrated across the upper GMS during the last millennium. Although no significant association was observed between gender and G6PD deficiency prevalence in the Lue population, there were much higher numbers of severe deficient males than females. Likewise, the enzyme activity of the deficient males was significantly lower than that of the deficient females (0.68 ± 0.62 U/g Hb in males and 3.17 ± 1.24 U/g Hb in females). Due to the fact that G6PD deficiency is an X-linked hereditary condition, males are usually affected more often than females, as males have only one X-chromosome^5,29^. Our observation was consistent with this scenario when all males who carried any type of G6PD variant displayed enzymatic deficiency. The G6PD enzyme activities in heterozygous females fluctuated among severe deficiency, mild deficiency, and normal activity levels. Being an X-linked trait, the expression of G6PD deficiency in heterozygote females was under Lyon’s theory. Owing to random inactivation of one of the two X chromosomes early in embryonic life, a mixture of normal and enzyme-deficient somatic cells can be found. The heterozygous females, thus, present the variability of expression depending on the level of the mosaicisms^30^.

The severity of the enzymatic activities of the members of the Lue population seemed to be caused by G6PD variations, but this was not consistent with the previous classifications of WHO Class II-severe or III-moderate-to-mild deficiency^27^. We observed that some Class II variation carriers exhibited moderate-to-mild levels of deficiency, while some of the Class III variation carriers exhibited a severe level of deficiency. Some scholars have pointed out the limitation of the WHO classification system in that it has often relied on a small number of observation and non-standardized laboratory methodology^31^. Moreover, the usage of an arbitrary cut-off value of 10% enzyme activity to distinguish between Class II and III variants was not found to be significantly associated with haemolytic severity in some research studies^1^. Incidences of contradictory co-occurrence between severe/moderate-to-mild deficiency levels and G6PD variants which were found in our present study and in some previous studies^13,31,32^ support the contention of revisiting or reclassifying the established WHO G6PD enzyme activity discrimination system. We also found 4 participants (1 hemizygote male and 3 heterozygote females) with mild enzyme deficiency but who could not be defined by their G6PD variation at any genotyped sites. There was a possibility that the deficiency-caused mutation was located out of the exon region. Mutations in intron splice sites and untranslated regions of genes have been reported to exert a reduction in protein levels through a defect in the transcription, splicing, translation, or gene regulation processes^33^. Effects of the variations in the non-exon parts on the G6PD enzyme activity still require further investigation.

In comparisons with the previous population-based G6PD prevalence reports, we found that prevalence levels of 14.28% in males and 12.82% in females observed in our Lue samples were higher than those of the populations located in Central Thailand (11.10% in males and 5.80% in females)^10^ and Southern Thailand (9.75% in males and 10.36% in females)^34^, but were nearly in the same range as those investigated in Northern Thailand (16.60% in males and 15.20% in females)^35^. Moreover, the prevalence in the Lue group was higher when compared with inhabitants of neighboring countries, i.e. Myanmar (11.60% in males and 9.60% in females), Laos (8.80% in males and 4.50% in females), Cambodia (26.10% in males and 3.10% in females) and Malaysia (5.30% in males and 1.05% in females)^12,23,36,37^. Interestingly, the common G6PD variation types found in the Lue group, Kaiping and Canton, were similar to those that were reported among the residences of the Northern Thailand and Southern China regions but were different from most of the populations in other parts of Thailand and Southeast Asia where the Viangchan and Mahidol variant types were found to be predominant^35,38,39^.

Our observation on the prevalence of G6PD deficiency, together with their gene variation patterns, revealed a genetic link between populations in Northern Thailand and Southern China. A high prevalence of G6PD Kaiping and Canton variants had been observed in the Northern Thai population, despite the inclusion of multiple ethnicities which may have introduced substantial genetic variabilities in the earlier study^35^. A similar distribution pattern had been previously reported among various ethnic groups residing in Southern China such as Dai, Zhuang, Yi^30^. Close genetic relationships between the Lue of Northern Thailand and the Dai ethnicity in Southern China, due to a shared line of ancestry, had been revealed by autosomal, Y-chromosomal and mitochondrial DNA markers^40–42^. Consistent with earlier findings, the G6PD distribution pattern observed in this study also supported a close affinity between the Lue and Dai people. However, it is worth noting that the G6PD Kaiping and Canton variants had also been found at high prevalence among the Sino-Tibetan speakers^38,39^. Although, the assimilation between the Chinese Han and Lue/Dai’s ancestor during the last millennium was evidenced^20,43^, their similar G6PD variation pattern could be due to the influenced of the same natural selection forces. Thus, we have proposed that the G6PD variation pattern found in the Lue population had been shaped ever since they were living in Southern China. And this pattern had been distributed across the upper GMS into the Northern part of Thailand as a consequence of the migration practices of these people.

Differentiations in the *G6PD *gene variations of the Lue and Northern Thai from the peoples in other regions of Thailand were particularly note-worthy. Several reports have investigated the predominance of G6PD Mahidol and Viangchan variants in people who have lived in Thailand, Myanmar Laos, Vietnam and Cambodia^10,14^. Subsequent ethnic-based studies have revealed the high prevalence of the G6PD Mahidol variant in several ethnic populations in the GMS, especially along the China-Myanmar and Thailand-Myanmar borders. This variant was observed in Kachin (22.00%), Mon (7.41%), Burmese (6.74%), Karen (13.84%), Jingpo (4.29%), and Burman ethnicities (8.06%)^11,13,23,31^. The G6PD Mahidol variant was purposed to be associated with the distribution of the *Plasmodium vivax* parasite under strong positive selection for the last 1500 years^44^. Interestingly, this G6PD Mahidol variant type was observed to be very rare in the Lue ethnic group. Several reliable historical documents have pointed out that the area identified as Xishuangbanna, Lue’s original homeland in Southern China, was one of the areas that was most endemic for malarial outbreaks. Incidences of malaria in Xishuangbanna, Southern China was 6000/10,000 in 1940 and then decreased to 16.81/10,000 in 2001^30^. Thus, regardless of the fact that the G6PD deficiency prevalence in Lue was as high as it was for the residents of the malarial endemic areas, they might be subject to other different selection forces from the G6PD Mahidol-predominant ethnic groups, and this could have resulted in different major G6PD types. Through the Lue’s unique genetic background and historical migration patterns that are known to be different from other ethnic groups of Thailand, we are assured that the Lue ethnicity provides a great opportunity to observe a new issue of different driving pressures in terms of the G6PD deficiency distribution in this country. Moreover, we have revealed that people in Thailand were not genetically homogenized. There were clearly distinctions in linguistic, cultural, and genetic roots of the people in different parts and ethnicities, as have been shown in a number of previous and this current studies^45,46^. The acquisition of accurate ethnic-based data on the prevalence of a hereditary disorder would therefore be necessary for the implementation of appropriate genetic counseling and prevention programs.

Although all Lue people located in northern Thailand share historic Dai ancestry, a comparison on the G6PD deficiency among six different Lue villages revealed a broad degree of prevalence, ranging from 8.33 to 17.95%. This is likely the result of the founder effect, a scenario in which a small number of members in an original population randomly migrates to a new locality. Since the thirteenth century, the Lue people of Xishuangbanna, Yunnan, Southern China were fragmented into several smaller groups and gradually migrated southward into more southern countries using different routes over different periods of time. They then carried randomly selected genetic resources from their ancestors and finally resettled with diverse genetic structures, as confirmed by our observations. We noticed a unique structure in the Lue5 population from Chiang Khong, Chiang Rai Province where only the G6PD Kaiping variant type was found in 10.64% of the samples. According to the village history, the main group of ancestors of these people migrated from Mueang Ou (literally meaning Ou city), which was previously known as the state of Xishuangbanna, China but now belongs to the Lao Republic. They then crossed the Mekong River via the Laos/Thailand border about 100 years ago and now reside at their present locality. Only one G6PD Kaiping predominance reflected a relatively strong drift effect in this population. In contrast, there were more diverse patterns of G6PD variations in other Lue villages. Apart from the G6PD Kaiping and Canton variants that were indicative of a genetic link between the Lue and Dai ethnic groups, as mentioned earlier, we could not specify the emergence of other types of variants in our Lue samples, i.e. the Union, Mahidol, Coimbra, Valladolid, Aures, and Gaohe variations. The Lue might have inherited these variants from their ancestors, and then frequency-shaped by the drift effect. Nevertheless, there was the possibility that the inter-ethnic admixture between the Lue and other ethnic groups might have occurred during the temporary settlement along their migration routes, such as with the Mueang Chiang Tung people, the Mueang Yong people in Myanmar or the Mueang Singh, Mueang Luang Nam Ta people of Laos. Unfortunately, reports on the G6PD deficiency prevalence and molecular characterization among the populations in Laos and Myanmar are very limited. Therefore, we were unable to clearly estimate the degree of admixture on the G6PD structure in members of the Lue ethnicity. Extended systematic studies on the prevalence and genetics of G6PD deficiency among the ethnic groups residing in the upper GMS countries could fulfill the existing knowledge gaps in terms of the evolutionary mechanisms associated with G6PD distribution, and may ultimately benefit public health planning programs.

## Conclusion

This study is the first ethnic-based report on the G6PD deficiency prevalence and molecular characterization in the Lue people of northern Thailand. The frequency of G6PD-deficient (13.51%, 40/296) and predominant G6PD Kaiping and Canton variations in the Lue people were more similar to those that had been previously investigated in the Northern Thai and ethnic residents of Southern China than of other Southeast Asian groups. This observation was in accordance with the evidence of shared ancestry between the Lue and Dai ethnic groups that was revealed in historical records and through earlier molecular evolutionary markers. However, a fluctuated level of prevalence among Lue villages reflects a difference in the degree of the founder effect in each population that occurred during the Lue people’s transboundary migration from Southern China to Northern Thailand hundreds of years ago.

## Supplementary Information

Supplementary Information
