# Supplementary material for: Prevalence and molecular characterization of glucose-6-phosphate dehydrogenase deficiency in the Lue ethnic group of northern Thailand
Source: Sci Rep. 2021 Feb 3;11:2956. doi: 10.1038/s41598-021-82477-w (PMC7858617; doi:10.1038/s41598-021-82477-w)
Supplement: Supplementary file 1 — Supplementary Information [file 41598_2021_82477_MOESM1_ESM.pdf]

# Prevalence and Molecular Characterization of Glucose-6-Phosphate Dehydrogenase Deficiency in the Lue Ethnic Group of Northern Thailand

Suwapat Sathupak[1,2] Kamonlak Leecharoenkiat[3] and Jatupol Kampuansai[1,4]*

[1]*Department of Biology, Faculty of Science, Chiang Mai University, Chiang Mai, Thailand*

[2] *Graduate School, Chiang Mai University, Chiang Mai, Thailand*

[3]*Oxidation in Red Cell Disorders Research Unit, Department of Clinical Microscopy, Faculty of Allied Health Sciences, Chulalongkorn University, Bangkok, Thailand*

[4] *Research Center in Bioresources for Agriculture, Industry and Medicine, Chiang Mai University, Chiang Mai, Thailand*

**Corresponding author: jatupol.k@cmu.ac.th*

**Supplementary Table 1** Primer sequences and exon of G6PD gene amplified

| Exon | Primer | Annealing Temp. (°C) | Size (bp) |
|---|---|---|---|
| 2 | 5'-CTCAAGAAAGGGGCTAACTTCTCAA-3'<br>5-'GGAATTCCTGGCTTTTAAGATTGGG-3' | 52 | 241 |
| 3, 4 | 5'-AGGATGATGTATGTAGGTCG-3'<br>5'- CCGAAGTTGGCCATGCTGGG-3' | 55 | 378 |
| 5 | 5'-GTGTGTCTGTCTGTCCGTGTC-3'<br>5-'CACGCTCATAGAGTGGTGGG-3' | 55 | 320 |
| 6, 7 | 5'-GGGAGGGCGTCTGAATGA-3'<br>5'-CCAGCCTCCCAGGAGAGAGG-3' | 55 | 572 |
| 8 | 5'-CATGCCCTTGAACCAGGTGA-3'<br>5'-GCATGCACACCCCAGCTC-3' | 54 | 241 |
| 9, 10 | 5'-TTCTCTCCCTTGGCTTTCTC-3'<br>5'-AAGACGTCCAGGATGAGGTGATC-3' | 51 | 737 |
| 11, 12, 13 | 5-'GAAGCCGGGCATGTTCTTCAAC-3'<br>5-'GTCAATGGTCCCGGAGTC-3' | 56 | 721 |

**Supplementary Table 2.** General data of G6PD deficiency prevalence among ethnic groups residing in the upper Greater Mekong Subregion for the purposes of comparison

| Ethnicity | Linguistic Family | No. of samples | No. of G6PD deficiency | | | Prevalence (%) | Reference |
|---|---|---|---|---|---|---|---|
| | | | Male | Female | Total | | |
| Mon1* | Austroasiatic | 162 | 19 | | 19 | 11.73 | Nuchphayoon (2008) |
| Khmer1 | Austroasiatic | 322 | 25 | 15 | 40 | 12.42 | Matsuoka (2005) |
| Khmer2 | Austroasiatic | 208 | 1 | 1 | 2 | 0.97 | Matsuoka (2005) |
| Khmer3 | Austroasiatic | 135 | 2 | | 2 | 1.48 | Matsuoka (2005) |
| Mon2* | Austroasiatic | 45 | 3 | | 3 | 6.67 | Iwai et al (2001) |
| Yao | Hmong–Mien | 3056 | 31 | 23 | 54 | 1.77 | Jiang et al. (2006) |
| Han | Sino-Tibetan | 133752 | 3084 | 2546 | 5630 | 4.21 | Jiang et al. (2006) |
| Hakka | Sino-Tibetan | 2607 | 81 | 27 | 108 | 4.14 | Jiang et al. (2006) |
| She | Sino-Tibetan | 1962 | 71 | 37 | 108 | 5.50 | Jiang et al. (2006) |
| Bai | Sino-Tibetan | 5292 | 43 | 23 | 66 | 1.25 | Jiang et al. (2006) |
| Yi | Sino-Tibetan | 442 | 18 | 5 | 23 | 5.20 | Jiang et al. (2006) |
| Jingpo | Sino-Tibetan | 192 | 19 | 12 | 31 | 16.15 | Jiang et al. (2006) |
| Hani | Sino-Tibetan | 495 | 23 | 13 | 36 | 7.27 | Jiang et al. (2006) |
| Ji Ruo | Sino-Tibetan | 322 | 46 | | 46 | 14.29 | Jiang et al. (2006) |
| Kachin (Jingpo) | Sino-Tibetan | 1770 | 187 | 336 | 523 | 29.55 | Li et al. (2015) |
| Kachin1 | Sino-Tibetan | 100 | 12 | 12 | 24 | 24.00 | Deng et al. (2017) |
| Jingpo | Sino-Tibetan | 372 | 12 | 10 | 22 | 5.91 | He et al. (2018) |
| Burmese1* | Sino-Tibetan | 178 | 17 | | 17 | 9.55 | Nuchphayoon (2008) |
| Burmese2* | Sino-Tibetan | 529 | 39 | | 39 | 7.37 | Iwai et al (2001) |
| Danu* | Sino-Tibetan | 28 | 2 | | 2 | 7.14 | Iwai et al (2001) |
| Kachin2* | Sino-Tibetan | 128 | 8 | | 8 | 6.25 | Iwai et al (2001) |
| Chinese* | Sino-Tibetan | 90 | 4 | | 4 | 4.44 | Iwai et al (2001) |

| | | | | | | | |
|---|---|---|---|---|---|---|---|
| Lisu* | Sino-Tibetan | 38 | 1 | | 1 | 2.63 | Iwai et al (2001) |
| Akha* | Sino-Tibetan | 45 | 0 | | 0 | 0.00 | Iwai et al (2001) |
| Zhaung | Tai-Kadai | 5255 | 338 | 156 | 494 | 9.40 | Jiang et al. (2006) |
| Dai1 | Tai-Kadai | 504 | 63 | 24 | 87 | 17.26 | Jiang et al. (2006) |
| Dai2 | Tai-Kadai | 1530 | 68 | 64 | 132 | 8.63 | He et al. (2018) |
| Shan* | Tai-Kadai | 37 | 4 | | 4 | 10.81 | Iwai et al (2001) |
| Lao* | Tai-Kadai | 291 | 21 | | 21 | 7.22 | Iwai et al (2001) |
| Lue | Tai-Kadai | 296 | 20 | 20 | 40 | 13.51 | Current study |

*data obtained from males only

**Supplementary Table 3.** G6PD enzyme activity and molecular typing of 58 G6PD variant carriers observed in the Lue population

| Population | Gender | G6PD unit (mU/ erytocyte per ml) | Hb (g/dL) | G6PD activity (U/g Hb) | G6PD typing | WHO group | Female (Homo/Hetero) |
|---|---|---|---|---|---|---|---|
| Lue5 | F | 60 | 13.3 | 0.45 | Kaiping | 2 | Hetero |
| Lue5 | F | 290 | 12.3 | 2.36 | Kaiping | 2 | Hetero |
| Lue6 | F | 350 | 13.7 | 2.55 | Coimbra | 2 | Hetero |
| Lue1 | F | 374 | 14.1 | 2.65 | Union | 2 | Hetero |
| Lue6 | F | 428 | 14.6 | 2.93 | Kaiping | 2 | Hetero |
| Lue2 | F | 365 | 11.5 | 3.17 | Kaiping | 2 | Hetero |
| Lue3 | F | 433 | 13.1 | 3.31 | Kaiping | 2 | Hetero |
| Lue1 | F | 431 | 12.1 | 3.56 | Union | 2 | Hetero |
| Lue1 | F | 540 | 13.1 | 4.12 | Union | 2 | Hetero |
| Lue5 | F | 667 | 14.7 | 4.54 | Kaiping | 2 | Hetero |
| Lue3 | F | 705 | 12.4 | 5.69 | Union | 2 | Hetero |
| Lue5 | F | 812 | 14.2 | 5.72 | Kaiping | 2 | Hetero |
| Lue3 | F | 769 | 13.2 | 5.83 | Kaiping | 2 | Hetero |
| Lue3 | F | 745 | 12.1 | 6.16 | Kaiping | 2 | Hetero |
| Lue2 | F | 847 | 13.2 | 6.42 | Kaiping | 2 | Hetero |
| Lue6 | F | 981 | 13 | 7.55 | Kaiping | 2 | Hetero |
| Lue5 | F | 930 | 11 | 8.45 | Kaiping | 2 | Hetero |
| Lue1 | F | 1031 | 11.2 | 9.21 | Union | 2 | Hetero |
| Lue3 | F | 1215 | 11.3 | 10.75 | Kaiping | 2 | Hetero |
| Lue1 | M | 60 | 15.3 | 0.39 | Valladolid | 2 | |
| Lue3 | M | 60 | 14.8 | 0.41 | Kaiping | 2 | |
| Lue6 | M | 60 | 13.9 | 0.43 | Union | 2 | |
| Lue1 | M | 60 | 12.6 | 0.48 | Valladolid | 2 | |
| Lue4 | M | 60 | 11.7 | 0.51 | Kaiping | 2 | |

| | | | | | | | |
|---|---|---|---|---|---|---|---|
| Lue3 | M | 108 | 13.8 | 0.78 | Union | 2 | |
| Lue2 | M | 249 | 14.5 | 1.72 | Kaiping | 2 | |
| Lue4 | F | 60 | 12.5 | 0.48 | Gaohe | 3 | Homo |
| Lue4 | F | 177 | 13.2 | 1.34 | Aures | 3 | Homo |
| Lue3 | F | 378 | 12 | 3.15 | Canton | 3 | Hetero |
| Lue6 | F | 563 | 15.2 | 3.70 | Canton | 3 | Hetero |
| Lue3 | F | 485 | 12.7 | 3.82 | Canton | 3 | Hetero |
| Lue1 | F | 536 | 13 | 4.12 | Viangchan | 3 | Hetero |
| Lue6 | F | 545 | 12 | 4.54 | Viangchan | 3 | Hetero |
| Lue4 | F | 651 | 13.7 | 4.75 | Canton | 3 | Hetero |
| Lue4 | F | 644 | 12 | 5.37 | Canton | 3 | Hetero |
| Lue6 | F | 796 | 13.3 | 5.98 | Canton | 3 | Hetero |
| Lue4 | F | 824 | 12 | 6.87 | Canton | 3 | Hetero |
| Lue3 | F | 747 | 9.7 | 7.70 | Canton | 3 | Hetero |
| Lue2 | F | 1128 | 12.5 | 9.02 | Canton | 3 | Hetero |
| Lue3 | F | 935 | 9.6 | 9.74 | Canton | 3 | Hetero |
| Lue6 | F | 1385 | 12.2 | 11.35 | Canton | 3 | Hetero |
| Lue2 | F | 1621 | 11.3 | 14.35 | Canton | 3 | Hetero |
| Lue2 | M | 16 | 14.2 | 0.11 | Canton | 3 | |
| Lue3 | M | 60 | 15.3 | 0.39 | Canton | 3 | |
| Lue6 | M | 60 | 15.3 | 0.39 | Viangchan | 3 | |
| Lue4 | M | 60 | 14.6 | 0.41 | Canton | 3 | |
| Lue3 | M | 60 | 14.1 | 0.43 | Canton | 3 | |
| Lue4 | M | 60 | 13.6 | 0.44 | Aures | 3 | |
| Lue4 | M | 60 | 13.2 | 0.45 | Gaohe | 3 | |
| Lue1 | M | 60 | 12.5 | 0.48 | Canton | 3 | |
| Lue3 | M | 60 | 12.2 | 0.49 | Canton | 3 | |
| Lue4 | M | 60 | 12.2 | 0.49 | Gaohe | 3 | |

| | | | | | | |
|---|---|---|---|---|---|---|
| Lue4 | M | 60 | 12 | 0.50 | Canton | 3 |
| Lue2 | M | 186 | 12.3 | 1.51 | Mahidol | 3 |
| Lue6 | F | 490 | 13 | 3.77 | Unknown variant | |
| Lue6 | F | 603 | 14.2 | 4.25 | Unknown variant | |
| Lue6 | F | 579 | 12.8 | 4.52 | Unknown variant | |
| Lue6 | M | 408 | 14.7 | 2.78 | Unknown variant | |
